# Influence of Filler Loading on the Mechanical Properties of Flowable Resin Composites

**DOI:** 10.3390/ma13061477

**Published:** 2020-03-24

**Authors:** Ioana-Codruţa Mirică, Gabriel Furtos, Bogdan Bâldea, Ondine Lucaciu, Aranka Ilea, Mărioara Moldovan, Radu-Septimiu Câmpian

**Affiliations:** 1 Department of Oral Health, Iuliu Hatieganu University of Medicine and Pharmacy, 400012 Cluj- Napoca, Romania; mirica_codruta@yahoo.com (I.-C.M.); ondineluc@yahoo.com (O.L.); arankailea@yahoo.com (A.I.); rcampian@email.com (R.-S.C.); 2 Department of Dental Materials, Babes-Bolyai University-Raluca Ripan, Institute of Research in Chemistry, 400294 Cluj-Napoca, Romania; mmarioara2004@yahoo.com; 3 Nicolae Testimiteanu, State University of Medicine and Pharmacy, 2004 Chisinau, Republic of Moldova; bogdanbaldea@gmail.com

**Keywords:** flowable resin composites, mechanical properties, compressive strength, flexural strength, flexural modulus

## Abstract

The aim of this study was to evaluate the correlation between the percent of inorganic filler by weight (wt. %) and by volume (vol. %) of 11 flowable resin composites (FRCs) and their mechanical properties. To establish the correlation, the quantity of inorganic filler was determined by combustion and shape/size analyzed by SEM images. The compressive strength (CS), flexural strength (FS), and flexural modulus (FM) were determined. The CS values were between 182.87-310.38 MPa, the FS values ranged between 59.59 and 96.95 MPa, and the FM values were between 2.34 and 6.23 GPa. The percentage of inorganic filler registered values situated between 52.25 and 69.64 wt. % and 35.35 and 53.50 vol. %. There was a very good correlation between CS, FS, and FM vs. the inorganic filler by wt. % and vol. %. (R^2^ = 0.8899–0.9483). The highest regression was obtained for the FM values vs. vol. %. SEM images of the tested FRCs showed hybrid inorganic filler for Filtek Supreme XT (A3) and StarFlow (A2) and a homogeneous type of inorganic filler for the other investigated materials. All of the FS values were above 50 MPa, the ISO 4049/2019 limit for FRCs.

## 1. Introduction

Flowable resin composites (FRCs) have been popular since 1995 because of their light cure and low viscosity. FRCs have a reduced filler content (37–53 vol. %) [1,2], which gives them a flowable character, spreading uniformly and adapting intimately to prepared tooth surfaces [2]. FRCs contain the same size of inorganic filler with conventional dental resin composites (DRCs) but a reduced content of inorganic filler [3,4] and a greater percent of diluent monomer [4]. Due to their low viscosity, FRCs have advantages such as easier handling properties during manipulation, better adaptation to the tooth surface, and higher flexibility [3]. FRCs can be placed in layers of minimum thickness to improve or eliminate air inclusion or entrapment, a particularly important property. Due to their high flexibility, there is a low probability that they will be displaced in stress concentration areas [1]. FRCs demonstrate radiopacity values above the minimum approved by the International Organization for Standardization (ISO) [5,6] and are available in different colors [1]. The drawbacks of FRCs include lower mechanical properties [3] and higher curing shrinkage [1]. Similar to conventional types of DRCs, the quantity, type, size of the inorganic filler [7], and the silanization of the inorganic filler significantly influence the mechanical properties [8]. The mechanical properties of FRCs depend on two main variables: the type of organic matrix [9,10], which gives the mechanical properties of the organic part [11,12], and the inorganic filler [13], characterized by the type, size, and geometry [13,14]. Other factors that influence the mechanical behavior of FRCs are the silanization [8], factors affecting the polymerization efficiency [15,16], and the binding between the inorganic filler and the organic matrix [8,17]. The clinical longevity of a dental material such as FRCs is correlated with the mechanical properties [18], because increased mechanical properties facilitate a good response to occlusal mechanical stress (clinical wear) [19]. 

The major clinical indications of FRCs are preventive resin restorations, pit and fissure sealants, cavity liners, Class V abfraction lesions, minimally invasive Class II restorations, and an inner layer for posterior Class II restorations [20]. The widespread use of these materials in an expanded range of applications places important demands on their mechanical properties [18]. The aim of this study was to evaluate the mechanical properties and correlation between the percent of inorganic filler by weight (wt. %) and by volume (vol. %) of eleven FRCs and their mechanical properties in the same testing conditions and by the same operator. The surfaces of FRC materials were investigated by a scanning electron microscope to establish the size and shape of the inorganic fillers in order to be correlated with the mechanical properties. The null hypothesis was that the percentage of fillers by weight and volume could be correlated with the mechanical properties of each FRC.

## 2. Material and Methods

### 2.1. Preparation of the FRC Samples for the Mechanical Test 

Samples (*n* = 8) from 11 commercially available FRCs were prepared. Table 1 shows the name of the FRC, the manufacturer, and the composition. The samples for the compressive strength (CS), were prepared by filling the FRC pasts in a cylindrical Teflon mold (8 mm in height and 6 mm in diameter) and pressing them between two glass slides covered with polyester strips. The polymerization was carried out using an XL3000 photocuring source (3M Dental Products, St Paul, MN, USA) for 60 s from the top and bottom. The samples were carefully removed from the mold after polymerization and the residual FRC material was removed from both surfaces by polishing with #800 and #1000 carbide papers under running water. Finally, the samples were stored in distilled water for 24 h at 37 °C before the tests were conducted. The flexural strength (FS), and flexural modulus (FM) tests were determined on parallelepipedic samples (*n* = 8) obtained in a Teflon mold with an internal dimension of 2 mm × 2 mm × 25 mm by curing them from the top and bottom in five 60-s stages along the length of the samples and storing the specimens as previously described. 

### 2.2. Percentage of Fillers by Weight and Volume

For each FRC, the inorganic filler content by wt. % and vol. % was calculated with the combustion analysis that was conducted in a furnace (HT 04-HT 450 Furnace, Nabertherm GmbH, Lilienthal, Germany). The specimens (*n* = 10) were dried at room temperature in a desiccator for 1 day before combustion and weighed to an accuracy of 0.00001 g. The combustion of the specimens was performed at 700 °C for 3 h. The resulting powder was stored for another day in the desiccator and weighed with the same accuracy. The percentage of inorganic fillers by weight was determined by investigating the differences in the weight of the specimens before and after the combustion. The inorganic filler weight percent was then transformed to a volume percent by applying the formula from Equation (1) [21].

(1) $$\mathrm{Fraction}\;\mathrm{of}\;\mathrm{fillers}\;\mathrm{vol}.\;\%=\frac{w_{f}/d_{f}}{w_{f}/d_{f}+w_{r}/d_{r}}\times 100$$

where *w_f_* and *w_r_* are the weight fractions of the inorganic filler and resin, the density of filler *d_f_* = 2.4 g/cm^3^, and the density of resin *d_r_* = 1.2 g/cm^3^ [22]. 

### 2.3. Mechanical Test 

The CS test was conducted by placing the samples in a universal testing machine (LR5K Plus, Lloyd Instruments, Ltd., Bognor Regis, England) and running the test at a speed of 0.75 mm/min until fracture. The speed 0.75 mm/min was chose in agreement with ISO 4049:2019 [23] where it is mentioned a load for the specimen at a cross-head speed of 0.75 ± 0.25 mm/min. The CS value (MPa) was calculated using the formula from Equation (2):

CS = F/π r^2^, (2)

where F is the applied load (N) and r is the radius of the cylindrical Teflon mold. 

**Figure 1:**
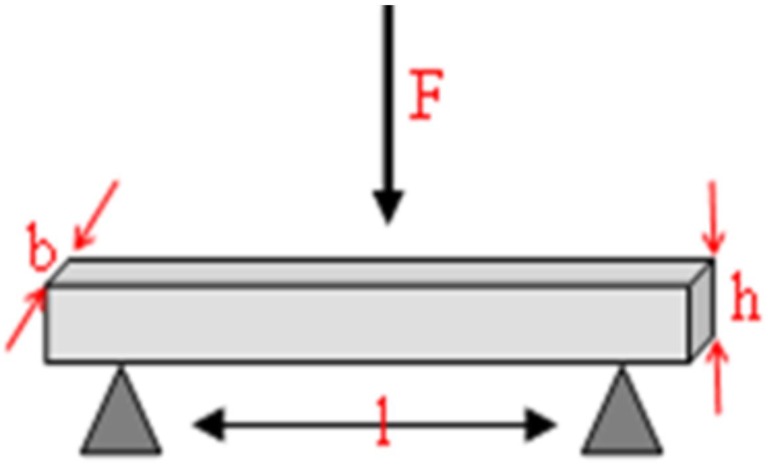
Schematic illustration of the flexural strength (FS) test.

The FS test was conducted using the 3-point bending test according to ISO 4049:2019 (Figure 1) [23]. The samples were placed on supports with a 20 mm span between them and the test was run at a speed of 0.75 mm/min until fracture. The FS values (MPa) were calculated using the following formula from Equation (3):

FS = 3F_max_l/2bh^2^, (3)

where F_max_ is the applied load (N), l is the span between the supports (20 mm), b is the width (2 mm), and h is the thickness (2 mm). 

The FM (GPa) was determined from the slope in the elastic portion of the stress–strain curve.

### 2.4. Scanning Electron Microscope (SEM) 

FRC disks of Accolade SRO (A2), Filtek Supreme XT (A3), ELS (Extra Low Shrinkage; A3 op), PermaFlo (A1), StarFlow (A2), and Wave (A3) measuring 8 mm diameter and 1 mm (±0.01) thickness were obtained by curing with an XL3000 photocuring source (3M Dental Products, St Paul, MN, USA) for 60 s. The FRC samples were polished using #800 and #1200 carbide paper. The specimen surfaces were evaluated by SEM (SEM Inspect S, FEI, Eindhoven, Netherlands) at an operating voltage of 15 kV and a magnification of 10.000×. 

### 2.5. Statistical Analyses 

The data were statistically evaluated using one-way analysis of variance (ANOVA) with SPSS (Version 11.5, SPSS, Chicago, IL, USA) software package, with a Tukey’s test with the level of significance set at 0.05 to calculate the significant differences between the mean values of the tested FRCs. 

## 3. Results

**Figure 2:**
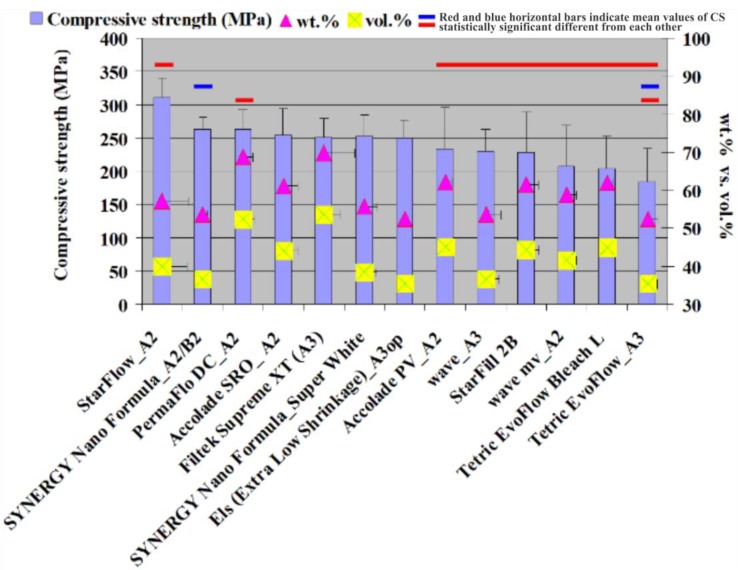
The results of CS (MPa; vertical error bars) and the filler percent by wt. % and by vol. % (horizontal error bars). Note: the red and blue horizontal bars indicate the mean values of CS were statistically significantly different from each other compared using a Tukey’s test, *p* < 0.05.

**Figure 3:**
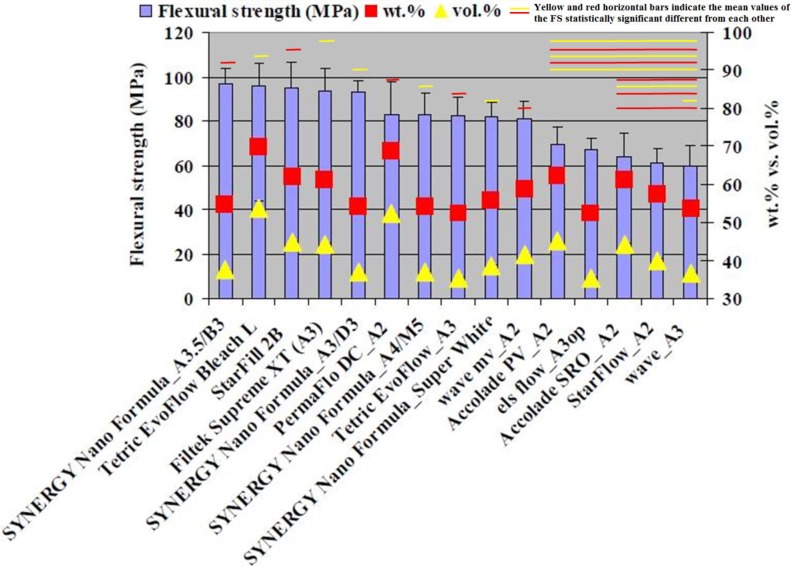
The results of FS (MPa; vertical error bars) and the filler percent by wt. % and by vol. % (vertical error bars). Note: the yellow and red horizontal bars indicate the mean values of FS were statistically significantly different from each other compared using a Tukey’s test, *p* < 0.05.

**Figure 4:**
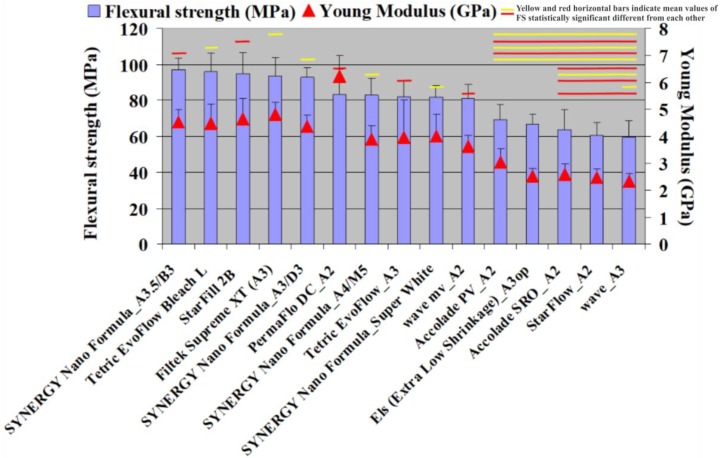
The results of FS (MPa) and flexural modulus (FM; GPa; vertical error bars). Note: the yellow and red horizontal bars indicate the mean values of the FS were statistically significant different from each other compared using a Tukey’s test, *p* < 0.05.

**Figure 5:**
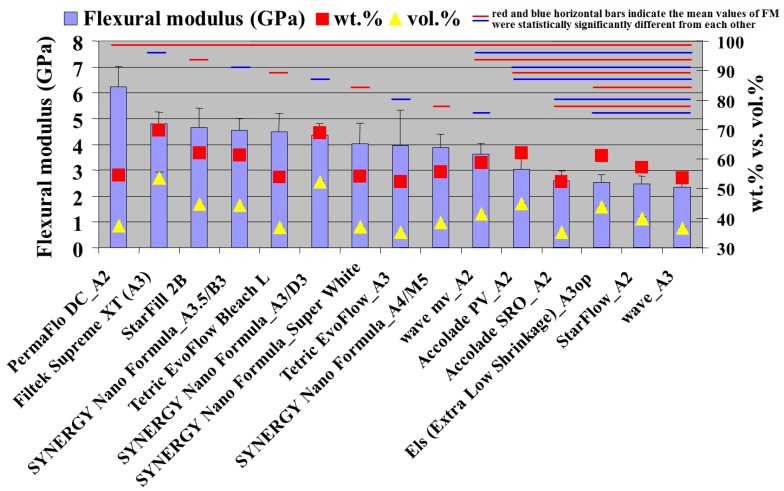
The results of FM (GPa; vertical error bars), filler percent by wt. % and by vol. %, Note: the red and blue horizontal bars indicate the mean values of FM were statistically significantly different from each other compared using a Tukey’s test, *p* < 0.05).

**Figure 6:**
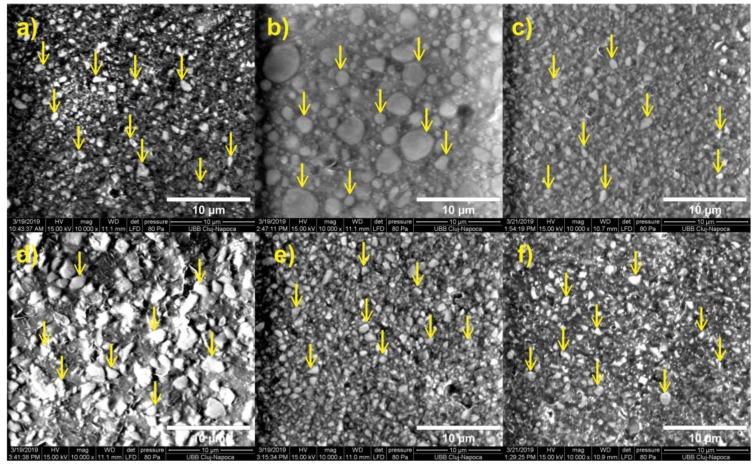
SEM micrographs of the FRC surfaces: (**a**) Accolade SRO (A2); (**b**) Filtek Supreme XT (A3); (**c**) ELS (extra low shrinkage; A3 op); (**d**) StarFlow (A2); (**e**) PermaFlo (A1); and (**f**) Wave (A3).

The combustion analysis that was conducted showed that the inorganic filler fraction ranged between 52.25 and 69.64 wt. % and 35.35 and 53.50 vol. % (Figure 2). The CS tests showed values between 182.87 and 310.38 MPa (Figure 2). The FS values were between 59.59 and 96.95 MPa (Figure 3). The FM test showed values from 2.34 to 6.23 GPa (Figure 4 and Figure 5). The SEM analysis of the FRC surfaces (Figure 6) showed a size mean of the inorganic filler between 0.67 and 1.67 µm. 

The SEM analysis of the FRC surfaces (Figure 6) showed a size mean of the inorganic filler of 0.67 (±0.17) µm for Accolade SRO (A2); 1.67 (±1.51) µm for Filtek Supreme XT (A3); 0.76 (±0.23) µm for ELS (Extra Shrinkage) (A3 op); 1.34 (±0.51) µm for StarFlow (A2); 0.94 (±0.24) µm for PermaFlo (A1); and 0.84 (±0.22) µm for Wave (A3).

## 4. Discussion 

The combustion analysis that was conducted showed that the inorganic filler fraction ranged between 52.25 and 69.64 wt. % and 35.35 and 53.50 vol. %. The data are presented in Figure 2. The literature [1,22] specified inorganic filler load values between 37 and 53 vol. % for FRCs. Almost all of the FRCs tested in our study showed values located within this range. An exception to this, with slightly lower values, is Tetric EvoFlow A3 (Ivoclar) with 35.38 vol. % and ELS (extra low shrinkage) A3 op (Saremco Dental AG) with 35.37 vol. %. Nitta et al. [24] reported values for inorganic FRC filler between 45.4 and 64.7 wt. % using the same method. The CS values ranged between 182.87 and 310.38 MPa (Figure 2). StarFlow A2 (Danville Materials) showed the best CS value and was statistically different (*p* > 0.05) from the materials with the lowest CS: Accolade PV A2 (Danville Materials), Wave A3 (SDI), Starfill 2B (Danville Materials), Wave mv A2 (Danville Materials), Tetric EvoFlow Bleach L (Ivoclar Vivadent), and Tetric EvoFlow A3 (Ivoclar Vivadent; Figure 2). The high CS result registered for StarFlow A2 (Danville Materials), an FRC with a decreased quantity of inorganic filler than other materials with lower CS values, could be explained by factors other than the quantity of inorganic filler, as previously mentioned [9,13,14]. The CS tests showed values between 182.87 and 310.38 MPa and were generally well correlated with the amount of inorganic filler, in agreement with another study [25]. The CS value is an important FRC property and can describe the endurance of the filling over time [26] because it is correlated with the forces developed during the masticatory process [8]. The FS was between 59.59 and 96.95 MPa (Figure 3). The best result was registered for SYNERGY Nano Formula A3.5/B3 (Coltene Whaledent) and was statistically different (*p > 0.05*) from the materials with lower FS values: Accolade PV A2 (Danville Materials), ELS (Extra Low Shrinkage) A3 op (Saremco Dental AG), Accolade SRO (Danville Materials), StarFlow A2 (Danville Materials), and Wave A3 (SDI; Figure 3).

The FS test was associated with the fracture behavior, which occurs more often in I, II, and III Class restorations [8]. Due to the limited clinical indications of FRCs, the ISO 4049/2019 standard [23] recommends a minimum FS value of 50 MPa for FRC materials. Our study showed FS results in concordance with the ISO 4049/2019 standard [23]. The values obtained in our study were also in agreement with the literature [13,27]. Recently a new FRC with TiO_2_ nanotubes fillers showed superior mechanical properties to FRC of unreinforced composite [28]. The FM test showed values from 2.34 to 6.23 GPa. The best value was registered for PermaFlo DC (Ultradent) and was statistically different (*p* > 0.05) from all of the other tested materials. The materials with the lowest FM values were StarFlow A2 (Danville Materials) and Wave A3 (SDI) (Figure 4). The FM results of our study were similar to other reports [11]. The FM results from our study were lower compared with the FM of the conventional DRCs and could be explained by the reduced amount of inorganic FRC filler [3]. Our results showed FM values in a linear correlation with the quantity of inorganic filler. This finding was in agreement with other studies from the literature [3,13]. The CS values and the inorganic filler by wt. % and vol. % of the FRCs were well correlated (R^2^ = 0.8899 and R^2^ = 0.8899). The highest correlation coefficients were also found for FS values and FM values vs. the values of inorganic filler by wt. % and vol. %. (R^2^ = 0.9483 and R^2^ = 0.9237). To calculate the correlation between the FM values vs. the inorganic filler values by vol. %, we excluded PermaFlo DC (A2; Figure 4). In our opinion, the high FM values (Figure 4) for PermaFlo DC (A2) could not only be influenced by the amount of inorganic filler, but also by the organic matrix. If we include PermaFlo DC (A2) when calculating the correlation coefficients between the FM values vs. the inorganic filler values by vol. %, we obtain a lower regression (R^2^ = 0.6699). The null hypothesis, that the percentage of fillers by weight and volume could be correlated with the mechanical properties was accepted. 

The SEM images (Figure 6) demonstrated a high difference between the inorganic fillers of Filtek Supreme XT (A3; Figure 6b), StarFlow (A2; Figure 6d), and all of the other investigated FRCs. These two materials had hybrid inorganic fillers with a mean size of the largest filler of 3.78 (±0.71) µm and the mean of the smallest filler of 0.77 (±0.48) µm. The yellow arrows from Figure 6 indicate fillers and shape of fillers at the surface of FRC. There was a smaller difference between the size of the particles from Filtek Supreme XT (A3) than StarFlow (A2; Figure 6b,e). The Filtek Supreme XT (A3) and StarFlow (A2) contained a hybrid inorganic filler. The inorganic filler in Filtek Supreme XT (A3) and StarFlow (A2) was a heterogenic type. This could explain the better mechanical properties for Filtek Supreme XT (A3) than Accolade SRO (A2); ELS (extra low shrinkage; A3 op); StarFlow (A2); and Wave (A3). An increased hybrid inorganic filler load improved the stress transfer at the interface of the particles in the FRCs [29,30]. In this study, Filtek Supreme XT (A3) showed very good mechanical properties, which could be explained by the use of hybrid inorganic fillers. An increase in the filler concentration diminished the mechanical properties because the particle–matrix adhesion strength decreases [31,32]. Even StarFlow (A2) had a hybrid inorganic filler and we could not see the same behavior like Filtek Supreme XT (A3). This could be attributed to other factors like mechanical properties of the polymer matrix, the degree of crosslinking, the type/quantity of silane, or the mechanical properties of the filler. For all of the other tested FRCs, the SEM images showed more homogenous types of inorganic filler. Only Filtek Supreme XT (A3) demonstrated spherical inorganic filler (Figure 6b). The nano- and microhybrid fillers showed high particle packing with a relatively low viscosity, improving workability, mechanical properties, and polish retention [33,34,35].

## 5. Conclusions

The percentage of inorganic filler registered was very well correlated with the mechanical properties and influenced the mechanical properties of the FRC. The highest regression was obtained for the FS values vs. the inorganic filler fraction vol. %. The tested FRC demonstrated FS values, which certificate them to be used as restorative materials as stated in ISO 4049/2019. The presence of hybrid inorganic fillers in the composition of some materials confirmed better mechanical properties. 

## Figures and Tables

**Table 1:** Flowable resin composites (FRCs) used in this study, information provided by the manufacturers.

No.	Product	Manufacturer	Shade	Composition
1	Accolade SRO	Danville Materials Inc. San Ramon, CA, USA	A2	65 wt. % inorganic filler, NC vol. %
2	Accolade PV	Danville Materials Inc. San Ramon, CA, USA	A2	65 wt. % inorganic filler, NC vol. %
3	Els (Extra Low Shrinkage)	Saremco Dental AG, Rebstein, Switzerland	A3op	Barium glass silanized, bis-GMA, bis-EMA, catalyst, inhibitors, and pigments; NC wt. %, NC vol. %
4	Filtek Supreme XT	3M ESPE, Seefeld, 3M ESPE	A3	bis-GMA, bis-EMA, TEGDMA, silica nanofiller, zirconia nanofiller and zirconia/silica nanocluster (65 wt. % inorganic filler, 55 vol. % inorganic filler)
5	PermaFlo	Ultradent, South Jordan, UT, USA	A1	Methacrylate monomer, alkylamino methacrylate, CQ, 68 wt. % inorganic filler, NC vol. %
6	PermaFlo DC	Ultradent, South Jordan, UT, USA	A2	70 wt. % inorganic filler, NC vol. %
7 8	Tetric EvoFlow	Ivoclar Vivadent, Schaan, Liechtenstein	A3 Bleach L	bis-GMA, UDMA, dimethacrylate, decandiol, prepolymers, additives, stabilizers and catalysts, pigments, barium glass filler, ytterbium trifluoride, silicon oxide, mixed oxide 57.5 wt. % inorganic filler, 30.7 vol. % inorganic filler
9	StarFill 2B	Danville Materials Inc. San Ramon, CA, USA		61 wt. % inorganic filler, 41 vol. % inorganic filler
10	StarFlow	Danville Materials Inc. San Ramon, CA, USA	A2	61 wt. % inorganic filler NC vol. %
11 12 13 14 15	SYNERGY Nano Formula	Coltene Whaledent, Altstaetten, Switzerland	Super White A2/B2 A4/M5 A3.5/B3 A3/D3	bis-GMA, Bis-EMA, TEGDMA, Strontium glass, Amorphous silica, Hydrophobed 55 wt. % inorganic filler 32 vol. % inorganic filler
16	wave	SDI, Bayswater, Vic, Australia	A3	35 wt. % multifunctional methacrylic ester 65 wt. % inorganic filler
17	wave mv	SDI, Bayswater, Vic, Australia	A2	40 wt. % multifunctional methacrylic ester 60 wt. % inorganic filler

bis-GMA: Bisphenol A diglycidylmethacrylate; bis-EMA: Bisphenol A polyethylene glycol diether dimethacrylate; UDMA: Urethane dimethacrylate; TEGDMA: Triethylene glycol dimethacrylate; CQ: Camphorquinone; wt. %: Percentage of filler by weight; vol. %: Percentage of filler by volume; NC: Information not collected.
